# Association between weight-adjusted-waist index and heart failure: Results from National Health and Nutrition Examination Survey 1999–2018

**DOI:** 10.3389/fcvm.2022.1069146

**Published:** 2022-12-14

**Authors:** Daoliang Zhang, Wenrui Shi, Zhaohui Ding, Jieun Park, Shaohui Wu, Jian Zhang

**Affiliations:** ^1^Department of Cardiology, Fuwai Hospital Chinese Academy of Medical Sciences, Shenzhen, China; ^2^Department of Cardiology, Shanghai Chest Hospital, Shanghai Jiao Tong University, Shanghai, China; ^3^School of Medicine, Shanghai Jiao Tong University, Shanghai, China; ^4^Heart Failure Center, State Key Laboratory of Cardiovascular Disease, Fuwai Hospital, National Center for Cardiovascular Diseases, Chinese Academy of Medical Sciences and Peking Union Medical College, Beijing, China

**Keywords:** epidemiology, heart failure, fat mass accumulation, weight-adjusted waist circumference index, obesity

## Abstract

**Background:**

Weight-adjusted waist circumference index (WWI) is a novel index positively associated with excessive fat accumulation. The current study aims to evaluate the association between WWI and the prevalent heart failure (HF), and to assess the value of WWI to improve the detection of HF in the general population.

**Methods:**

A total of 25,509 subjects from National Health and Nutrition Examination Survey 1999–2018 were included into our study. WWI was calculated as WC (cm) divided by the square root of weight (kg). HF was identified according to the subjects’ reports.

**Results:**

The prevalence of reported HF was 2.96%. With adjustment of demographic, anthropometric, laboratory, and medical history data, one SD increment of WWI could cast an additional 19.5% risk for prevalent HF. After separating WWI into quartiles, the fourth quartile had a 1.670 times risk of prevalent HF compared to the first quartile. Furthermore, smooth curve fitting suggested that the association was linear in the entire range of WWI. Moreover, the association was robust to subgroups of age, sex, race, obesity, hypertension, and diabetes. Additionally, ROC analysis revealed a significant improvement for the detection of prevalent HF from WWI (0.890 vs. 0.894, *P* < 0.001); And continuous net reclassification index (0.225, *P* < 0.001) and integrated discrimination index (0.004, *P* < 0.001) also supported the improvement from WWI.

**Conclusion:**

Our data demonstrated a significant, linear, and robust association between WWI, a simple surrogate for fat mass accumulation, and the risk for prevalent HF in a representative population. Moreover, our results also suggested the potential value of WWI to refine the detection of prevalent HF in the general population.

## Introduction

Heart failure (HF) is a complicated syndrome developed at the end stage of various cardiovascular diseases (1). The prevalence of HF remains a continuous rising trend, and is estimated to be more than 37.7 million individuals globally (2). Until 2011, an estimated 5.7 million patients suffered from HF and 870,000 new cases were diagnosed per year in the United States (3). In many developing countries, the burden of cardiovascular diseases, including HF, is also under a rapid increasing stage (4). Under this grim situation, it is essential to improve the early diagnosis of HF in the general population.

Obesity is one of the major risk factors for the development of HF, both heart failure with persevered ejection fraction (HFpEF) and heart failure with reduced ejection fraction (HFrEF) (5). In the Framingham Heart Study, obesity was identified to independently associate with HF risk with the adjustment of other cardiovascular risk factors (6). Later, the association between obesity and HF risk was confirmed in a study with larger population (7). Furthermore, a similar association between increased body mass index (BMI) and the risk of HF was also observed in non-US population (8, 9). More importantly, distribution of adiposity could play a pivotal role in the impact of obesity on HF incidence and prevalence; a magnetic resonance imaging study demonstrated that visceral but not subcutaneous adipose tissue was related to adverse cardiac remodeling (10). However, the widely used indicator of obesity, BMI and waist circumference (WC), were unable to differentiate between muscle mass and fat mass (11).

In 2018, Park et al. proposed a novel anthropometric index named weight-adjusted-waist index (WWI) (12). WWI has been demonstrated to positively correlated with fat mass but negatively associated with muscle mass (13). Later studies have identified that WWI is associated with cardiovascular diseases and mortality (14–16). However, the association between WWI and the prevalent HF remains unclear. Therefore, the current study aims to evaluate the association between WWI and the risk of prevalent WWI, and to assess whether WWI could improve the detection of prevalent HF in the general population.

## Materials and methods

### Subjects

Our current analysis was a secondary analysis of the National Health and Nutrition Examination Survey (NHANES) 1999–2018. The NHANES survey refers to a series of cross-sectional survey conducted by the National Center for Health Statistics (NCHS), an affiliated department of the Centers for Disease Control and Prevention. The survey was conducted in the United States for every 2 years in the past two decades. The survey adopted a multistage, stratified, and clustered probability sampled pattern to maintain its representativity. The data from different circle of survey could be appended together for integrated analysis. More detailed information about the NHANES design and conduction is available to the public at the NHANES official website.^1^ In the current analysis, the inclusion criterion was subjects aged ≥ 20 and ≤85 years old. The exclusion criteria were subjects with incomplete data used in the current analysis. A total of 25,509 subjects were finally enrolled into our study (Supplementary Figure 1). The protocol of NHANES survey was approved by the NCHS institutional Ethics Review Board, and our current study did not contain any person identifying material. Therefore, the current study did not require further ethic review. All data used in our study could be downloaded from the NHANES official website.

### Data collection and measurements

Interviews were conducted at subjects’ home during the data collection process, and laboratory examinations were performed at the Mobile Examination Center (MEC). Demographic data were collected by trained stuffs through a computer-assisted interviewing system. For subjects who could not answer the question by themselves, a family member would answer the questions instead. Drink for at least 12 times during the past year before enrollment was regarded as current drinking. Subjects answered “some days” or “every day” to the question “Do you now smoke cigarettes” were determined as current smoking subjects. The poverty-to-income ratio (PIR) was used to estimate the socioeconomic status, and it was calculated by family income ratio to the federal poverty threshold. The definition of HF is based on the question “Someone ever told you had congestive heart failure” from the questionnaire. Similarly, coronary heart disease (CHD) was defined as answering “yes” to the question “Someone ever told you had coronary heart disease.”

Anthropometric parameters were collected with a standard operation procedure. Waist circumference (WC) and height were quantified to the nearest 0.1 cm; weight was quantified to the nearest 0.1 kg. Blood pressure was measured after sitting and resting quietly for at least 5 min. The mean value of three blood pressure recordings was used in the current analysis. More detailed information about the blood pressure measurement is available in the “Physician Examination Procedures Manual” on the NHANES official website.

Laboratory examinations were conducted at laboratories certified by the CDC. Fasting plasma glucose (FPG) was measured by the oxygen rate method on the Modular Chemistry side of the Beckman DxC800; Blood lipids were quantified by enzymatic assay on the Roche modular P and Roche Cobas 60,000 chemistry analyzers. Serum creatinine (Scr) was determined by DxC800 modular chemistry side through the Jaffe rate method.

### Definition

Body mass index was calculated as weight (kg) ratio to height (m) squared. Answering “Yes” to the question “Take diabetic pills to lower blood sugar” or “Taking insulin now” was regarded as anti-diabetic therapy; FPG ≥ 7 mmol/L and/or self-reported use of anti-diabetic therapy was defined as diabetes (17). Answering “Yes” to the question “Now taking prescribed medicine for hypertension” was determined as anti-hypertensive therapy; A mean systolic blood pressure (SBP) ≥ 140 mmHg, a mean diastolic blood pressure (DBP) ≥ 90 mmHg, and/or anti-hypertensive therapy were indicated as hypertension (18). Answering “Yes” to the question “Now taking prescribed medicine for cholesterol” during the interview was defined as lipid-lowering therapy. WWI was calculated as WC (cm) divided by the square root of weight (kg) (12).

### Statistical analysis

In our study, statistical data were weighted because of the survey design of NHANES.^2^ Categorical variates were also summarized as frequency and 95% CI. Continuous variates were listed as the mean value with 95% confidence intervals (CI). Comparison of categorical variates and continuous variates were conducted by Chi-square test and *t*-test, respectively. Association between WWI and the risk of prevalent HF was assessed by multivariate logistic regression analysis. The results were displayed as odds ratios (ORs) and 95% CI. Furthermore, A generalized addictive model with a spline smooth-fitting function was conducted to investigate whether the association was linear in the entire range of WWI. Finally, receiver-operating characteristic (ROC) curve and reclassification analysis (continuous net reclassification index, NRI and integrated discrimination index, IDI) were conducted to assess the potential value of WWI to improve the detection of prevalent HF. All the statistical analysis was conducted by s Stata Statistical Software (version 15.0; StataCorp. LLC., College Station, TX, USA), statistical software packages R^3^ (The R Foundation) and EmpowerStats^4^ (X&Y Solutions, Inc., Boston, MA, USA). A two-tailed *P*-value less than 0.05 was regarded as statistical significance.

## Results

### Subjects characteristics

The characteristics of enrolled subjects were summarized in Table 1. The prevalence of HF was 2.96% (754/25509). Regarding the demographic data, HF patients had a significantly higher age level, larger percentages of male sex, white race, and current drinking status, and a subsequently lower PIR level than subjects without HF. For the anthropometric data, weight, BMI, WC, SBP levels were significantly higher in HF patients, while DBP level was lower in HF patients. Laboratory exams revealed that FPG, total cholesterol (TC), and Scr were higher in HF patients and HDL-c was higher in subjects without HF. Medical history data showed that the percentages of receiving anti-hypertensive therapy, anti-diabetic therapy, lipid-lowering therapy, and CHD history were remarkably higher in HF patients than their counterparts. Accordingly, the prevalence of hypertension and diabetes were subsequent lower in subjects without HF. Finally, the level of WWI was significantly higher in HF patients than in subjects without HF.

**TABLE 1:** Subjects’ characteristics.

Variables	Total (25,509)	Reported HF (*n* = 754)	Without reported HF (*n* = 24755)	*P*-value
Age (years)	46.77 (46.18–47.26)	65.66 (64.41–66.90)	46.34 (45.85–46.83)	< 0.001
Male (%)	48.84 (48.28–49.39)	54.82 (50.18–59.38)	48.70 (48.13–49.28)	0.006
Race (%)				0.013
Non-hispanic white	73.60 (71.16–75.90)	78.90 (75.59–81.87)	73.48 (71.02–75.80)	
Non-hispanic black	8.50 (7.54–9.55)	6.54 (4.47–9.46)	8.54 (7.58–9.61)	
Mexican American	9.54 (8.28–10.96)	9.90 (7.78–12.52)	9.53 (8.27–10.96)	
Other hispanic	6.74 (5.52–8.22)	3.30 (2.01–5.35)	6.82 (5.58–8.31)	
Others	1.63 (1.35–1.95)	1.36 (0.52–3.54)	1.63 (1.36–1.96)	
Current drinking (%)	26.73 (25.41–28.10)	32.79 (29.28–36.50)	26.60 (25.26–27.97)	< 0.001
Current smoking (%)	17.36 (16.48–18.28)	18.46 (15.23–22.21)	17.34 (16.43–18.29)	0.548
PIR	3.02 (2.94–3.09)	2.29 (2.13–2.45)	3.03 (2.96–3.10)	< 0.001
Height (cm)	168.98 (168.78–169.18)	167.33 (166.41–168.25)	169.02 (168.81–169.22)	0.119
Weight (kg)	82.36 (81.89–82.82)	89.40 (86.87–91.94)	82.20 (81.74–82.65)	< 0.001
BMI (kg/m^2^)	28.76 (28.59–28.93)	31.81 (30.92–92.71)	28.69 (28.53–28.85)	< 0.001
WC (cm)	98.76 (98.33–99.19)	109.51 (107.56–111.45)	98.51 (98.09–98.93)	< 0.001
SBP (mmHg)	121.97 (121.59–122.35)	129.08 (127.33–130.84)	121.80 (121.43–122.18)	< 0.001
DBP (mmHg)	70.43 (70.02–70.83)	66.53 (65.33–67.74)	70.51 (70.11–70.92)	< 0.001
FPG (mmol/L)	5.47 (5.44–5.51)	6.58 (6.32–6.84)	5.45 (5.42–5.48)	< 0.001
TC (mmol/L)	5.07 (5.04–5.09)	4.65 (4.51–4.78)	5.07 (5.05–5.10)	< 0.001
HDL-c (mmol/L)	1.40 (1.38–1.41)	1.26 (1.22–1.29)	1.40 (1.39–1.41)	< 0.001
Scr (μmol/L)	78.78 (78.31–79.26)	105.33 (99.83–110.82)	78.18 (77.73–78.64)	< 0.001
Anti-hypertension therapy (%)	25.59 (24.61–26.61)	72.06 (67.84–75.91)	24.54 (23.59–25.53)	< 0.001
Anti-diabetic therapy (%)	6.92 (6.53–7.34)	29.81 (25.79–34.16)	6.41 (6.03–6.80)	< 0.001
Lipid-lowering therapy (%)	16.04 (15.31–16.80)	53.35 (48.32–58.31)	15.20 (14.47–15.96)	< 0.001
Hypertension (%)	32.73 (31.68–33.79)	77.85 (73.70–81.50)	31.71 (30.68–81.50)	< 0.001
Diabetes (%)	10.91 (10.39–11.45)	36.92 (32.30–41.80)	10.32 (9.82–10.84)	< 0.001
CHD history (%)	3.28 (2.97–3.63)	40.74 (36.11–45.53)	2.44 (2.17–2.73)	< 0.001
WWI (cm/√kg)	10.94 (10.91–10.96)	11.66 (11.58–11.75)	10.92 (10.90–10.94)	< 0.001

Data were summarized as mean (95% confidence intervals) or numbers (95% confidence intervals) according to their data type.

HF, heart failure; PIR, poverty-to-income ratio; BMI, body mass index; WC, waist circumference; SBP, systolic blood pressure; DBP, diastolic blood pressure; FPG, fasting plasma glucose; TC, total cholesterol; HDL-c, high density lipoprotein cholesterol; Scr, serum creatinine; CHD, coronary heart disease; WWI, weight-adjusted waist circumference.

### Linear association between weight-adjusted waist circumference index and the prevalent heart failure

Logistic regression analysis was conducted to assess the association between WWI and the prevalent HF. The results were showed in Table 2. In the crude model, each SD increase of WWI could cast a 2.484 times risk of prevalent HF. After adjustment of demographic covariates, including age, sex, race, current smoking and drinking status, and PIR, the additional risk for each SD increase of WWI diminished to 66.9%. With the further adjustment of BMI, WC, Scr, FPG, TC, high-density lipoprotein (HDL), SBP, anti-hypertensive therapy, anti-diabetic therapy, lipid-lowering therapy, and CHD history, the additional risk reduced to 19.5%. When separating WWI values into quartiles, the risk for prevalent HF increased significantly alone with the elevation of WWI quartiles (*P* for trend = 0.040), and the top quartile had a 1.670 times risk of prevalent HF when compared to the first quartile.

**TABLE 2:** Association between weight-adjusted waist circumference index (WWI) and the reported heart failure (HF).

Variables	Odds ratio (95% CI)					
	Crude	*P*-value	Model 1	*P*-value	Model 2	*P*-value
WWI (Per SD increase)	2.484 (2.265–2.725)	< 0.001	1.669 (1.472–1.893)	< 0.001	1.195 (1.036–1.379)	0.015
Quartiles of WWI						
Quartile 1	Reference		Reference		Reference	
Quartile 2	3.222 (2.153–4.821)	< 0.001	1.883 (1.255–2.825)	0.003	1.361 (0.877–2.111)	0.167
Quartile 3	7.171 (5.197–9.896)	< 0.001	2.763 (1.942–3.931)	< 0.001	1.545 (1.027–2.323)	0.037
Quartile 4	15.231 (10.873–21.335)	< 0.001	4.039 (2.781–5.865)	< 0.001	1.670 (1.059–2.634)	0.028
*P* for trend		< 0.001		< 0.001		0.040

Crude: no adjustment; Model 1: age, sex, race, current smoking, current drinking, PIR; Model 2: model 1 + BMI, WC, Scr, FPG, TC, HDL-c, SBP, anti-hypertensive therapy, anti-diabetic therapy, lipid-lowering therapy, and coronary heart disease history.

**FIGURE 1:**
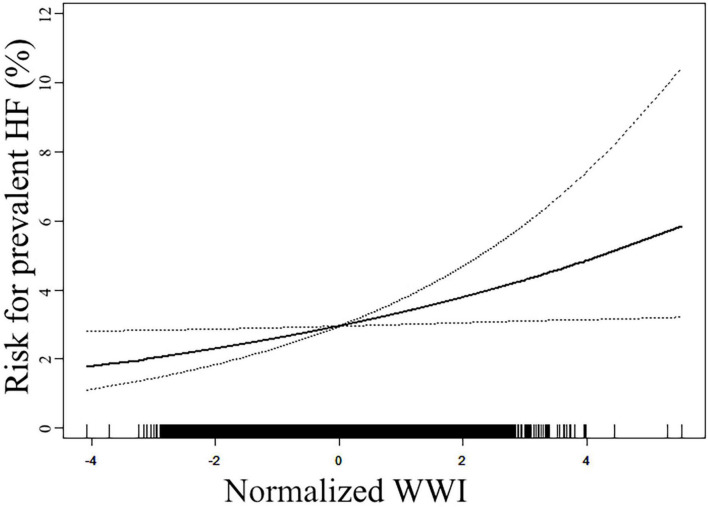
Smooth curve fitting to evaluate the linearity of the association between weight-adjusted waist circumference index (WWI) and the reported heart failure (HF). The model was adjusted for age, sex, race, current smoking, current drinking, poverty-to-income ratio (PIR), body mass index (BMI), waist circumference (WC), serum creatinine (Scr), fasting plasma glucose (FPG), total cholesterol (TC), high-density lipoprotein (HDL), systolic blood pressure (SBP), anti-hypertensive therapy, anti-diabetic therapy, lipid-lowering therapy, and coronary heart disease (CHD) history (The same as Model 2 in Table 2). The solid line in the plot referred to the estimated risk of prevalent reported HF, and the dotted lines indicated the pointwise 95% CI. The association followed a linear pattern in the entire range of WWI.

Our study also employed a generalized additive model with a smooth curve fitting function to assess the linearity of the association between WWI and the prevalent HF (Figure 1). The model was adjusted for all covariates used in Model 2 of Table 2. The results displayed that the association was linear in the entire range of WWI. The risk for prevalent HF increased from around 2% at the lowest end of WWI to more than 4% at the highest end of WWI.

### Robustness of the association between weight-adjusted waist circumference index and the prevalent heart failure

**FIGURE 2:**
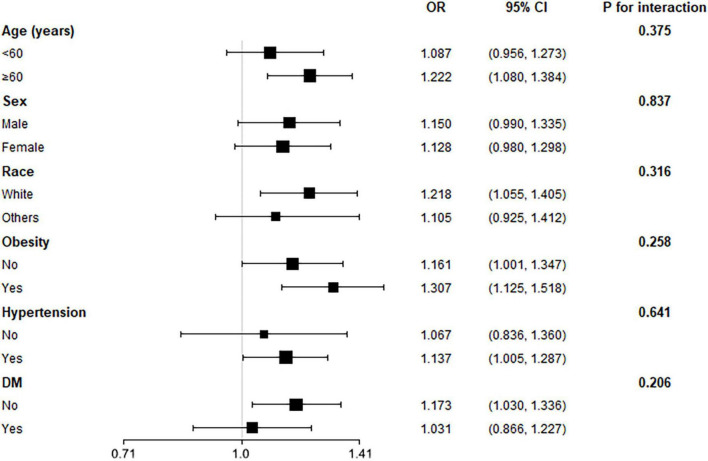
Subgroup analysis for the association between weight-adjusted waist circumference index (WWI) and the prevalent reported heart failure (HF). The multivariate logistic model adjusted for all variates in used in Model 2 of Table 2, except for the variate that used to define subgroups. None of the stratified variables, including age, sex, race, obesity (BMI ≥ 30 kg/m^2^), hypertension, and diabetes, significantly modified the association between WWI and the reported HF (all *P* for interaction > 0.05).

To investigate whether the association between WWI and the prevalent HF was robust among several conventional cardiovascular subpopulations, we conducted subgroup analysis with interaction test (Figure 2). The model was adjusted for all covariates in Model 2 of Table 2, except for the covariates used to define subgroups. The figure showed that our main finding was robust in subgroups of age (<60 or ≥60), sex, race (white or others), obesity, hypertension, and diabetes, with all *P* for interaction > 0.05.

### Significant value of weight-adjusted waist circumference index to refine the detection of prevalent heart failure

Receiver-operating characteristic and reclassification analysis were employed to assess the value of WWI to refine the detection of prevalent HF (Table 3 and Supplementary Table 1). The AUC of WWI for the detection of prevalent HF was 0.709 (0.704–0.715), significantly higher than that of BMI (0.598, 95% CI: 0.592–0.604) and WC (0.659, 95% CI: 0.653–0.665). When adding WWI into conventional risk factors (including BMI and WC), the AUC for prevalent HF significantly increased from 0.890 (0.886–0.893) to 0.894 (0.890–0.897). About the reclassification analysis, both continuous NRI (0.225, 95% CI: 0.152–0.297) and IDI (0.004, 95% CI: 0.002–0.007) revealed a significant improvement for the detection of prevalent HF when adding WWI into conventional cardiovascular risk factors.

**TABLE 3:** Receiver-operating characteristic (ROC) and reclassification analysis for weight-adjusted waist circumference index (WWI) to improve the identification of reported heart failure (HF).

Model	AUC (95% CI)	*P*-value	*P* for comparison	NRI (continuous)	*P*-value	IDI	*P*-value
WWI	0.709 (0.704–0.715)	< 0.001	−	–	–	–	–
BMI	0.598 (0.592–0.604)	< 0.001	< 0.001	–	–	–	–
WC	0.659 (0.653–0.665)	< 0.001	< 0.001	–	–	–	–
Clinical risk factors*	0.890 (0.886–0.893)	< 0.001	−	–	–	–	–
Clinical risk factors + WWI	0.894 (0.890–0.897)	< 0.001	0.002	0.225 (0.152–0.297)	<0.001	0.004 (0.002–0.007)	0.002

*Clinical risk factors: age, sex, race, current smoking, current drinking, PIR, BMI, WC, Scr, FPG, TC, HDL, SBP, anti-hypertensive therapy, anti-diabetic therapy, lipid-lowering therapy, and coronary heart disease history.

## Discussion

In the current study, our data demonstrated a significant and positive association between WWI, a novel, easy-acquired indicator of adipose obesity, and the prevalent HF in a representative American population. Furthermore, the association was linear in the entire range of WWI, suggesting risk of prevalent HF increased proportionally with the elevation of WWI. Moreover, the association was robust in several conventional cardiovascular subpopulations. Finally, results from both ROC and reclassification analysis demonstrated the significant value of WWI to refine the detection of prevalent HF. Therefore, our analysis suggests the potential association between WWI and the risk of prevalent HF in the general population, and our findings also support the potential incremental value of WWI to optimize the detection of prevalent HF in the general population.

Our results confirmed the association between WWI and the risk of prevalent HF in the general population. In multivariate logistic regression analysis, WWI showed a significant and positive association with the prevalent HF even after adjusting for demographic, anthropometric, laboratory, and medical history covariates. It is necessary to mention that the adjusted covariates included BMI and WC. Therefore, the association between WWI and prevalent HF is independent from the confounding effect of general obesity and simple abdominal obesity, suggesting the impact of fat mass on the risk of prevalent HF. Furthermore, we also observed a significant and linear trend toward higher risk of prevalent HF. Moreover, smooth curve fitting also confirmed the linear trend; the risk for prevalent HF increased proportionally with the elevation of WWI value, without any threshold or saturation effect. These results implicate that WWI could serve as an independent and linear indicator for the risk of prevalent HF in the general population.

The results from the subgroup analysis demonstrated that the significant association between WWI and the prevalent HF was consistent among several conventional cardiovascular subpopulations. Results displayed in Figure 2 revealed that the association was robust to age, sex, race, obesity, hypertension, and diabetes subgroups. The ORs in these subgroups were similar to the OR observed in the entire population. Some subgroups showed difference, such as sex subgroups, race subgroups, and obesity subgroups; However, the none of the *P*-value for interaction reached statistical significance. Importantly, we observed a difference in OR value between subjects with and without obesity. Subjects with obesity had a higher risk increase for prevalent HF for each SD increase of WWI than subjects without obesity, suggesting obesity subjects could be more vulnerable to the increase of WWI, and the underlying fat mass. However, the difference did not achieve statistical significance. Hence, this finding still requires more study to validate.

Receiver-operating characteristic and reclassification analysis revealed the potential role of WWI in detecting prevalent HF. The AUC of WWI was significantly higher than that of BMI and WC, suggesting the superior value of WWI in HF identification. Furthermore, when introducing WWI into conventional cardiovascular risk factors, we observed a significant improvement for the identification of prevalent HF. Nevertheless, ROC analysis still has its limitation even if it is the most common approach to assess the diagnosis ability of novel markers. ROC analysis possesses a low sensitivity to identify the usefulness of a new index to improve the risk identification of prevalent diseases (19). Rather than detecting the value of an index itself to improve risk identification, ROC only compares the ability of different models (20). Therefore, ROC analysis alone may be insufficient to evaluate the impact of a new index to refine the risk identification of prevalent diseases. Accordingly, statisticians have proposed reclassification analysis to investigate the incremental value of new indexes for refining risk identification of prevalent diseases (21–23). In the current analysis, both continuous NRI and IDI were significant, implicating a significant and incremental value of WWI to optimize the risk identification of prevalent HF. In general, both ROC and reclassification analysis reported an incremental value of WWI to refine the risk identification of prevalent HF. Therefore, clinicians may achieve more precise identification of patients with high risk of prevalent HF from the general population by applying WWI into primary care settings.

Our findings were consistent with established data. In a recent published article, Huynh et al. demonstrated that increased intramuscular thigh muscle fat accumulation is independently associated with HF, suggesting excessive adiposity deposition, especially the intramuscular fat, contributes to the elevation of HF risk (24). From the view of pathophysiology, excessive adipose accumulation could lead to HF through multiple pathways, including altered hemodynamics, cardiac structure remodeling, inflammation, and neurohumoral and cellular dysfunction (25). Our current study suggests that WWI, a simple estimate of fat mass, may associate with the risk of prevalent HF. Therefore, our study provides a clue to transform the pathophysiological association between fat accumulation and HF into clinical practice.

Except of some novel findings, our study still has some disadvantages. First, the cross-sectional design of the NHANES did not allow us to assess the value of WWI to predict the occurrence of HF. Therefore, the goal of our current study focused on the association between WWI and the presence of HF, and we also aimed to evaluate whether WWI could improve the detection or identification of the presence of HF in the general population. Studies with longitudinal design is required to confirm our findings. Second, we excluded a number of NHANES participants due to lack of related variates, which could introduce bias into our results. Third, some variables, such as the medication history and the self-reported HF, relied on the recall of the participants, and we could not categorize the subtypes of HF. This could introduce information bias into our current study. However, these variables are naturally difficult to define according to objective criteria in large sample size studies, and the NHANES was conducted strictly to its protocol. Therefore, we believe the information bias was still acceptable in our current study, but we still need more studies to confirm our findings. Fourth, the NHANES study was conducted only in the United States, whether our findings are applicable to other populations remains unclear. Last, we have adjusted for demographic data, anthropometric data, laboratory data, and medication data in our current study. Nevertheless, many factors associated with HF could confound the association between WWI and HF. However, due to the limited data provided by NHANES, we could not adjust all confounders in the current study. This is a natural limitation of observation studies. Accordingly, more studies with more detailed data collection are needed to validate our results. Based on above four points, more longitudinal studies with more detailed information are needed to confirm our findings.

## Data availability statement

Publicly available datasets were analyzed in this study. The dataset supporting the conclusions of this article is available from the corresponding authors on appropriate request. All the data could be downloaded from the NHANES official website.

## Ethics statement

The NCHS Institutional Ethics Review Board approved the study protocol of NHANES. The patients/participants provided their written informed consent to participate in this study.

## Author contributions

DZ and WS designed the current study. WS, ZD, and JP integrated and analyzed the data. DZ, WS, and SW drafted the manuscript. JZ revised the manuscript and proofread it for publication. All authors contributed to the article and approved the submitted version.

## Footnotes

1
https://www.cdc.gov/nchs/nhanes/index.htm

2
https://wwwn.cdc.gov/nchs/nhanes/tutorials/module3.aspx

3
http://www.R-project.org

4
http://www.empowerstats.com
