# Supplementary material for: Association between weight-adjusted-waist index and heart failure: Results from National Health and Nutrition Examination Survey 1999–2018
Source: Front Cardiovasc Med. 2022 Dec 14;9:1069146. doi: 10.3389/fcvm.2022.1069146 (PMC9794568; doi:10.3389/fcvm.2022.1069146)
Supplement: Supplementary file 1 [file Data_Sheet_1.docx]

**Supplemental Materials**

**Figure S1.** Flow chart of the subjects enrolling process.

NHANES enrolled a total of 101316 participants from 1999 to 2018. Among them, 48010 subjects were excluded because they aged ≤ 20 or ≥ 85. 27428 subjects were dropped due to missed data of HF. Moreover, 369 subjects were further excluded due to missed data of related covariates. Finally, a total of 25509 subjects were enrolled into the current analysis.


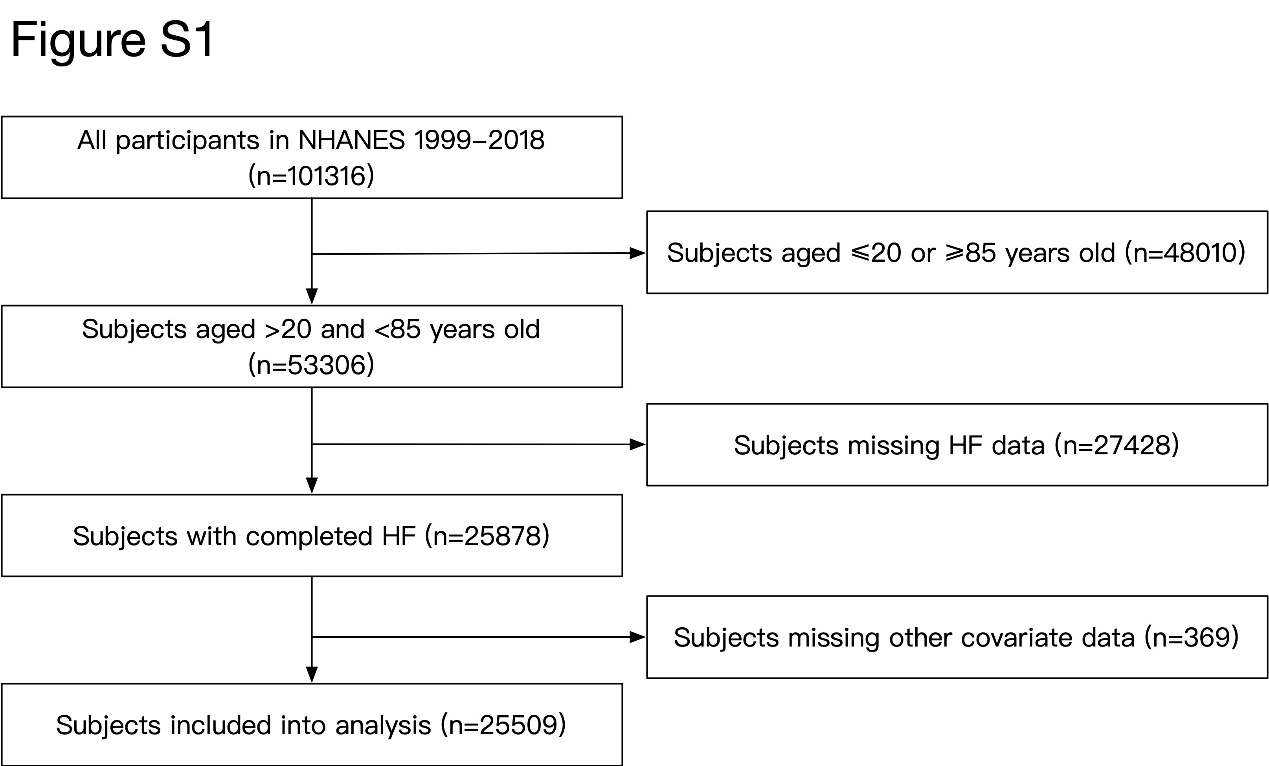


**Table S1.** Details of the ROC analysis for WWI to identify reported HF.

| Index | AUC (95% CI) | P value | P for comparison | Sensitivity (%) | Specificity (%) | NPV (%) | PPV (%) |
| --- | --- | --- | --- | --- | --- | --- | --- |
| WWI | 0.709 (0.704-0.715) | <0.001 | - | 77.6 | 54.9 | 98.8 | 49.8 |
| BMI | 0.598 (0.592-0.604) | <0.001 | <0.001 | 51.5 | 63.6 | 97.7 | 41.3 |
| WC | 0.659 (0.653-0.665) | <0.001 | <0.001 | 71.5 | 52.4 | 98.4 | 43.8 |

Abbreviations: ROC: receiver operating curve; WWI: weight-adjusted-waist index; HF: heart failure; AUC: area under the curve; NPV: negative predictive value; PPV: positive predictive value
